# Protocol for the collection, packaging, and transportation of preterm newborn stool samples

**DOI:** 10.1016/j.clinsp.2024.100531

**Published:** 2024-11-13

**Authors:** Jessica Santos Passos Costa, Heli Vieira Brandão, Mara Viana Cardoso Amaral, Gabriela Cintra dos Santos, Camilla da Cruz Martins, Michelle de Santana Xavier Ramos, Tatiana de Oliveira Vieira, Raquel Guimarães Benevides, Graciete Oliveira Vieira

**Affiliations:** a Health Research and Extension Center, Postgraduate Program in Public Health, Department of Health, Universidade Estadual de Feira de Santana, Feira de Santana, BA, Brazil; b PhD in Public Health, Universidade Estadual de Feira de Santana, Feira de Santana, BA, Brazil; c PhD in Medicine and Human Health, Escola Baiana de Medicina e Saúde Pública, Salvador, BA, Brazil; d Health Research and Extension Center, Department of Health, Universidade Federal do Recôncavo da Bahia, Cruz das Almas, BA, Brazil; e Master's degree in Public Health, Universidade Estadual de Feira de Santana, Feira de Santana, BA, Brazil; f PhD in Medicine and Health, Universidade Federal da Bahia, Salvador, BA, Brazil; g Department of Biology, Universidade Estadual de Feira de Santana, Feira de Santana, BA, Brazil; h PhD in Biologie Structurale et Nanobiologie, Université Joseph Fourier ‒ Grenoble I, France

**Keywords:** Microbiota, Colostrum, Preterm newborn, Clinical protocols

## Abstract

**Background:**

The newborn's intestinal microbiota can vary with gestational age. Reliable analyses of stool samples require establishing the steps of collection, packaging, transportation, and storage.

**Objective:**

To describe the development of a protocol and test an algorithm for the sequence of actions and procedures for the collection, packaging, transportation, and storage of stool samples from Preterm Newborn (PTNB) admitted to a Neonatal Intensive Care Unit (NICU).

**Materials and methods:**

This is a descriptive, observational study linked to a non-randomized controlled clinical trial conducted in the NICU of two public hospitals covenanted to the Unified Health System of a large city in inland northeastern Brazil. The methodology adopted in its development followed the Brazilian Ministry of Health Preparation Guide recommendations.

**Results:**

The flow of actions and procedures was consistent with scientific evidence. The sequence of work process steps to implement the proposed protocol was tested and resulted in a graphical representation of an algorithm compatible with the reality of Brazilian public hospitals.

**Conclusion:**

Following the protocol steps with the description of the rules of conduct and recommendations regarding the collection, packaging, transportation, and storage of stool samples from PTNBs ensured the preservation and integrity of the bacterial DNA in the stools sample. The details of the recommendations will allow their reproducibility and improvement by professionals and researchers with similar study objects.

**Trial registration:**

World Health Organization (WHO) under Universal Trial Number (UTN) code U1111–1266–2295, under register RBR-3mm7cs in the Brazilian Registry of Clinical Trials (REBEC).

## Highlights

• The steps prior to the fecal metagenomic analysis of premature infants are detailed.
• Surgical tissue added to the diaper prevents absorption and preserves the microbead.
• Hygienize the neonate after collection, pay attention to its comfort and vital signs.
• The protocol guaranteed the preservation bacterial DNA in 78 % of the samples.

## Introduction

There is a growing interest in studying the human microbiome, which consists of the collection of genes housed in microbiota cells that can interact with the genome.^1^ This interaction is established since the beginning of life and influences the health-disease process. It may be associated with morbidity in the long term^,^ such as asthma, obesity, and neurological development disorders.^2^, ^3^, ^4^ In the neonatal period, the authors highlight the clinical importance of the intestinal microbiota for the health of Preterm Newborns (PTNB) in preventing necrotizing enterocolitis, a pathology with high neonatal mortality, which affects 42 % of PTNBs with extremely low weight.^5^^,^^6^^,^^7^

The most studied microbiota to date is the bowel, composed of diverse populations including bacteria, fungi, viruses, and protozoa, which are modulated by genetic predisposition, lifestyle, and exposure type, that begins during pregnancy. The biodiversity of this microscopic community grows during the breastfeeding period, and the microbiota established in childhood tends to stabilize at the end of adolescence. Several factors can affect the human milk microbiota and, thus, the intestinal microbiome of the newborn, such as maternal factors: pre-gestational Body Mass Index (BMI), delivery type, use of antibiotics, weight gain during pregnancy, maternal diet, genetics, health, demographic or environmental differences, pump use for breast expression; and neonatal factors: use of antibiotics, food type, delayed enteral feeding, delayed or limited physical contact with the mother, prolonged stay in neonatal intensive care, prematurity, active infection and contact with the infant oral cavity.^2^^,^^8^, ^9^, ^10^, ^11^ Furthermore, external factors such as differences in DNA collection, extraction, and sequencing techniques may contribute to variations in identifying the milk microbiota and the intestinal microbiota.^8^

Regarding the diet type, the establishment of healthy intestinal microbiota in the newborn can be influenced by breastfeeding and the immunological modulation promoted by this practice.^12^, ^13^, ^14^, ^15^ Although human milk was initially considered a sterile fluid and isolated microorganisms were considered external contaminants, it is currently accepted that it has its own microbiota. The widely accepted hypothesis for the origin of these microorganisms in human milk is the entero-mammary route, which allows the transfer of bacteria from the maternal intestine via immune, dendritic, and CD18 cells to the mesenteric lymph nodes and, finally, to the mammary gland.^8^^,^^16^^,^^17^

There is also evidence that specific human milk bacteria can be transferred to the newborn's intestine and influence the general composition of the intestinal microbiota, with lasting implications for health in the short and long term.^2^^,^^10^^,^^14^^,^^18^ Knowledge of this microbiota can help promote the health of PTNBs and prevent adverse clinical outcomes.

Knowing the intestinal microbiota is a complex task, and the collecting PTNB stool samples is a challenge since this population has several peculiarities and specificities that limit its access and handling. Moreover, properly packaging and transporting stool samples with a cold chain is paramount for preserving and identifying genetic material.^19^

This study aimed to develop an innovative protocol and algorithm with a graphic representation of the sequence of actions and procedures for collecting, packaging, and transporting fecal samples from PTNBs admitted to a Neonatal Intensive Care Unit (NICU). It is worth mentioning that it was observed that all publications in this area do not mention in detail techniques for rescuing/collecting fecal samples from preterm infants, although they describe well the sequencing and analysis methods. This bias implies comparability between study results.

## Materials and methods

This is a descriptive, observational study linked to a controlled, non-randomized, superiority clinical trial entitled “Metagenomic Analysis of the Intestinal Microbiota of Premature Infants on Oropharyngeal Colostrum Immunotherapy Care Attended at the Health Unic System (SUS): An Intervention Study” (our free translation from Portuguese), conducted in a large city in the Brazilian Northeast. The methodology used in the current study followed the recommendations of the Brazilian Ministry of Health Guide to Clinical Protocols and Therapeutic Guidelines.^20^

### Characterization of study sites and the population

The study was conducted in two public hospital units linked to the Unified Health System (SUS), which provides services to women (during pregnancy, labor, childbirth, and puerperium) and newborns.^21^^,^^22^

The primary population presumed by the recommendations of this protocol consisted of PTNBs with birth weight ≤ 1,500 g, gestational age ≤ 36 weeks, and admitted to the NICU. The inclusion criteria were PTNB who started the OCI fasting and later were on an exclusive human milk diet or remained fasting and clinically stable.

Exclusion criteria were a) For the mother – maternal history of psychoactive substance use, drugs, alcohol, psychological disorders, multiple gestations from triplets and children of mothers contraindicated for breastfeeding (retroviruses); b) For the newborn – use of vasopressor medication > 10 mcg/Kg/min, a requirement for immediate surgical intervention, syndromes or congenital malformations, sepsis, or necrotizing enterocolitis. For testing this protocol, 50 newborns were included by convenience sampling.

### Protocol steps

The steps that constitute the protocol are the collection, storage, and transport of stool samples. Before collection, family members and/or companions, when present, were informed about the procedures that would be carried out. The collector, properly dressed and with sanitized hands, organized the necessary material close to the patient and in the order in which it would be used to collect the stool sample.

### Collection of stool samples

A cutout of a (fenestrated and sterile) surgical drape fabric was placed inside the diaper of the preterm at birth for stool collection, which covered the entire internal part, to prevent the content (stool sample) from being immediately absorbed by the diaper. The indicated dimensions of the fabric cutout were 15 cm long by 5 cm wide, the size that best fit inside the diaper (proportions adopted after running tests with different sizes).

Furthermore, placing the sterile tissue prevents the absorbent part of the diaper, impregnated with barium salts, from creating a bias in the results since these are stool pathogen inhibitors.^23^^,^^24^ New surgical drape cutouts were replaced inside the diaper in all newborn changes by the technicians who were in routine procedures.

When the newborns defecated, the diaper was carefully removed by the team of nurses and duly dressed so that there was no contamination of the stool sample. Then, routine hygiene was performed on the PTNBs. The intervals between changes occurred per the team's routine (approximately every 3 h).

The surgical drape cutout containing the sample (positioned inside the closed diaper) was collected with a sterile Ayre spatula. The content was placed directly in the 1.5-mL sterile Eppendorf tube with an airtight seal. A total of 0.75 mL of the stool sample were collected, approximately half of the tube.

### Packaging and storage of stool samples

The microtubes containing the collected fecal material were placed in a plastic storage rack to prevent the Eppendorf from tipping over and immediately sent for the first freezing in an ordinary freezer (253,15k, −20 °C in Portuguese) located in the hospital unit, with a digital thermometer with an extension cable for temperature control.

The sample remained for a maximum of 12 h in the hospital's freezer to preserve the material's durability and quality. Then, it was transported to an ultra-freezer (193,15k, −80 °C in Portuguese) located in the Laboratory of Microbiological Analysis at UEFS, where the sample would remain frozen until the process of extracting and sequencing the bacterial DNA of the samples.

### Transportation of stool samples

The transportation of the (frozen) tube from the hospital freezer to the State University of Feira de Santana (UEFS) ultra-freezer was carried out in an isothermal, hygienic, and impermeable container, with a digital thermometer with an extension cable attached to the box to control the temperature of the transported material, thus preserving the cold chain.

Moreover, to preserve cooling, hard reusable ice (gelox) was used for transportation at low temperatures, thus avoiding sample thawing at some point. The gelox was kept in a freezer for approximately 30 h before being used for transportation.

### Evaluation of potential risks

A pilot plan was initially implemented to assess potential risks, feasibility, steps for implementing the routine suggested through the practical protocol, and research biases to prepare this practical orientation. Therefore, it was necessary to perform on-site team training to implement the protocol correctly. Meetings were held with sector professionals. Videos, explanatory leaflets, and flowcharts were presented, and the team of researchers monitored the initial collections to ensure that there were no doubts.

The team of researchers closely observed the sequential progression of actions during this step, and feedback from professionals was obtained through open-ended questions about the process. There was no need to change the algorithm of the proposed actions, even with the reported implementation difficulties.

In the care of PTNBs, to minimize risks, light movements were recommended while placing the diaper and the surgical drape cutout. For the closure, care was taken not to put pressure on the belly, not to make the diaper tight, and not to leave too much volume between the legs, avoiding discomfort. Furthermore, diaper changes must be quick, and the caregiver must pay attention to the PTNBs’ signs of discomfort and loss of heat or agitation. The baby's hygiene was only performed after collecting the samples to avoid contamination.

### Ethical issues

The research was approved by the Research Ethics Committee of the State University of Feira de Santana (UEFS) under CAAE number: 16,995,219.0.0000.0053 and by the Brazilian Registry of Clinical Trials under UTN: U1111-1248-6732.

Mothers of PTNBs were invited to participate in the research within 24 h after delivery, with support from the psychology service. During the conversation, clarifications were given about the data collection procedures and the importance of intensive care and nutrition for the recovery of the PTNBs. The research was conducted upon acceptance to participate (by signing the Informed Consent Form). Once inserted in the research, a notice was placed on the incubator with an alert for the collection of stool samples.

## Results

The results of this research are the presentation of the algorithm with the specific recommendations of the flowchart steps and the confirmation of the feasibility of the proposed method in preserving the diversity of the fecal microbiota after metagenomic analysis.

### Algorithm

**Fig. 1:**
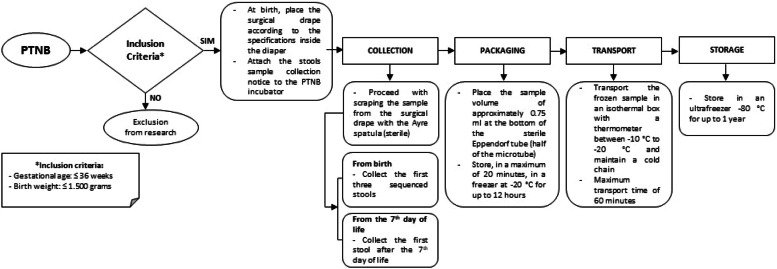
Algorithm of the stool sample collection, packaging, transportation, and storage protocol for extracting genetic material, 2022.

Besides presenting the protocol in text form, a graphical representation was performed as an algorithm, defining the finite sequence of the work process flow for the collection of stool samples, packaging, transportation, storage, and extraction of genetic material (Fig. 1). The development of this tool was grounded on operational protocols for health services, international publications, and guidelines from the book by Werneck (2009).^25^^,^^26^ The protocol was improved during the health team's work routine through daily practice while implementing the steps.

### Flowchart steps’ recommendations

Currently, there are no systematized evidence-based recommendations in the literature. Thus, it is strongly recommended that the present practical protocol be developed for the test public of the study in which the algorithm was tested, PTNBs admitted to the NICU. Table 1 presents the recommendations that cover each step of the flowchart of actions of this protocol regarding the resources and the materials used for the development of the protocol.

**Table 1:** Recommendations for each stage of the protocol flowchart, Feira de Santana, 2021.

**Collection**	**At birth ‒** Fix the notice on the incubator, signaling to the team that the newborn was included in the research. With each collected stool, an “X” must be marked on the notice attached to the incubator with a permanent pen, indicating that collections have been performed.
	**Definition of surgical drape cutout ‒** The tissue can be any brand, as long as it is fenestrated and sterile. Surgical tissue is sold in different sizes; however, the specifically recommended cutout must be made by the team of researchers and adapted to the size of the diaper used in the NICU routine. Thus, for the dimension tests, the researchers will require using personal protective equipment (cap, glove, mask, and apron), besides metal scissors duly disinfected with 70 % alcohol. The cutouts must be stored in airtight-sealed ZipLock plastic bags (disinfected with 70 % alcohol) to avoid contamination.
	**Recommended field dimensions –** The tissue should be cut 15 cm long and 5 cm wide. Although this protocol recommends specific dimensions, the cutout can be adapted to the size of the diaper used in the sector.
	**Care when placing the surgical drape ‒** Recommended sizes should be followed to prevent the tissue from sticking out of the diaper or that it is too small, not ensuring the collection of the entire sample. Another recommendation is that the side of the tissue in contact with the newborn should be the toweled one (since the fabric has two sides, plastic and toweled).
	**Spatulas –** A sterile plastic Ayre spatula individually packaged and opened only at the time of use is recommended.
	**Microtubes ‒** Sterile Eppendorf centrifuge tube, 1.5 mL.
	**Rack box for tubes ‒** A rack box to store the microtube with a capacity of 100 cryogenic tubes from 1.5 mL to 2.0 mL with a hinged lid is recommended.
	**Collection time ‒** After defecation, the collection must occur within a maximum of 3 h (following the flow of the hospital) so as not to occur total absorption of the sample by the diaper.
	**Personal protective equipment (PPE) –** Only professionals wearing PPE (cap, gloves, mask and hospital gown) should change diapers to ensure the quality of the material collected.
	**Collection ‒** The diaper must be opened on a clean surface properly disinfected with 70 % alcohol (such as a counter for example). The rack containing the microtubes and the Ayre spatula must be opened similarly. Then, the fecal material is collected and deposited in the microtube. The tube must be sealed and sanitized (70 % alcohol), encoded (with a permanent pen), and sent to the domestic freezer (253,15k) within 10 min.
	**Research Box –** The neonatal sector should have a plastic box with a lid 25.8 cm long, 17.8 cm wide, and 8.5 cm high to store the material for the fecal collection described above.
**Packaging**	**Freezer ‒** A (vertical or horizontal) freezer with a freezing capacity of up to 253,15k should stand next to the neonatal unit to store the samples (for a maximum of 12 h).
	**Thermometer ‒** The freezer temperature must be controlled using a digital thermometer with maximum and minimum temperatures with an external sensor and alarm.
	**Rack box for tubes –** A rack box for microtube storage inside the freezer should be available to prevent the tubes from turning over.
**Transportation**	**Thermal box ‒** The transportation of the (frozen) tube from the hospital freezer to the UEFS ultra-freezer must occur in an isothermal, hygienic, and waterproof container, with a digital thermometer with an extension cord attached to the box to control the temperature of the transported material, thus preserving temperature from 263,15k to 253,15k. 5–6-liter plastic cooler 20.8 cm long, 21.3 cm wide, and 28.3 cm high should be used.
	**Gelox –** Hard reusable ice (gelox) was used for transport to preserve the cooling of stool samples at low temperatures, thus avoiding sample. Gelox was kept in a freezer for approximately 30 h before being used for transportation. Two 400 mL, 17 cm long, 10 cm wide, and 2.7 cm high gelox units were used to transport the samples.
	**Rack box for tubes –** A rack box for storing the microtubes inside the thermal box should be available to prevent the tube from turning over.
	PPE should always be used for handling the tube.
**Storage**	**Ultra-freezer ‒** Permanent freezing should be realized in an ultra-freezer at 193,15k. These precautions preserve the genetic material.
	**Rack box for tubes –** A rack box for storing the microtubes inside the ultra-freezer should be available to prevent the tubes from turning over. PPE should always be used when manipulating the Eppendorf tube.

### Feasibility and fecal microbiota diversity

The use of this protocol made it possible to obtain preserved genetic material from fecal samples for metagenomic analysis, with a high count of DNA reads (< 1000 reads), through sequencing of the PTNB 16S rRNA gene. Of the 100 samples analyzed, 78 showed integrity of the bacterial DNA, a fact that made it possible to identify genera of aerobic and anaerobic bacteria.

## Discussion

The studies published to date have made little mention of the collection, transport and storage of fecal samples, fundamental criteria to guarantee the quality of the extracted material and enable the genomic sequencing of the microbiota contained in the samples. The lack of control over these procedures can contribute to heterogeneity between results and compromise comparability between studies.^27^^,^^28^ Problems and failures in the collection of biological material are serious and can compromise the diagnosis and the quality of care provided. This protocol adopted an algorithm defining the flow of the healthcare team's work process, with strict measures to control and preserve the quality of DNA from fecal samples, with the aim of minimizing risks or losses to the extraction of genetic material and future metagenomic analyses.^28^^,^^29^

Care with the material to be analyzed must occur from the first stage of the process. The collection of fecal samples inside the PTNB incubators with sterile instruments and immediate packaging in sterilized microtubes reduces the risk of contamination, as it guarantees their safety, with a reduction in external factors that could eventually contaminate the sample (environment external to the incubator, handler's hand, inadequate storage temperature), and interfere with the type of microbial species identified, as at birth the newborn's microbiota is quickly influenced by the external environment.^2^^,^^30^

Still on fecal collection, an innovative aspect of the current protocol was the detailed description of the careful retrieval of fecal samples through the use of surgical drape tissue (fenestrated and sterile), which covered the internal part, preventing the fecal content from being absorbed. by the diaper or that could be contaminated by its components. In an investigation carried out in China, which aimed to improve the efficiency of extracting bacterial DNA from the meconium of newborns, it was found that, in part of the samples obtained by scraping directly from the diaper, fragments were found (traces of cellulose fibers, superabsorbent polymer, among other components that make up its composition), which blocked the analysis filter column and compromised DNA extraction efficiency.^30^

Other essential points to be considered in protocols that require cryopreservation are the time and storage conditions until freezing and the ultra-freezing process. Thus, in the second step (packaging), as soon as they were collected, the samples were frozen within 10 min in a domestic freezer (253,15k), Relevant caution, as evidence shows that the bacterial composition of feces changes after 15 min of remaining at room temperature.^30^

These samples remained frozen for a maximum of 12 h until they were transported to the laboratory, an essential criterion to interrupt the biological processes of degradation and cellular damage, generated by the interruption of the cold chain. Stool sample freezing (193,15k or 253,15k) immediately after collection, a procedure adopted in the current protocol, is considered the gold standard for microbiota studies, especially when using metagenomic analysis.^27^

When transporting stool samples to packaging in the laboratory's ultra-freezer, it was essential to manage the cold chain, avoiding unwanted thawing. To this end, they were transported in cooling boxes with essential elements to preserve the temperature, such as ice packs and recording devices like digital thermometers.^29^ This measure is of great value, especially for places where the ultra-freezer is not located in the same area as fecal sample collections, meaning safe transport is necessary.^29^

The stool samples were received at the laboratory, identified, and immediately frozen in an ultra-freezer at 193,15k in the storage step. Cryopreservation media are in everyday use due to their ability to ensure feasibility and cell activity recovery, a fundamental step in sequencing-based technologies to determine the microbiome. However, the standardization of methods guides technicians and researchers. It enables more robust and comparable conclusions between studies involving collecting biological material for DNA/RNA extraction or any other experimental work to study the microbiome.^19^ Thus, the advantages of following a standard protocol with previously tested steps are reiterated.^29^

Care in all stages of this protocol made it possible to preserve the integrity of the bacterial DNA of 78 fecal samples from the PTNB, a fact that allows us to conclude that this was successful, as it was possible to accurately measure the microbiota of the collected samples and minimize changes in taxonomic compositions.^30^

This study stands out for its pioneering spirit in developing a protocol for collecting fecal samples from PTNBs and in defining an algorithm to guide the flow of the health team's work process in a public hospital in the Northeast of Brazil, which can be used by health professionals or researchers in any circumstances that aim to preserve the quality of the collected material and the integrity of the bacterial DNA, in order to identify species in the intestinal tract. The research results can be generalized to other public or private health services. Furthermore, it will undoubtedly contribute to the reduction of risks and the safety of patients and health professionals involved in the care of preterm infants and their mothers, as well as contributing to the consolidation of this knowledge and comparability of results.

The main limitation in the development of the current protocol was the precariousness of robust studies on the collection, packaging, transport and storage of fecal samples from preterm infants admitted to neonatal intensive care units, which would guide the construction of the algorithm steps, a limitation that gave it an innovative characteristic of the present study.

Other limitations refer to difficulties during the implementation of the protocol, such as issues related to practical application with regard to the adaptation and integration of professionals to the stages of the new clinical practice flow. It was necessary to carry out training and continuing education, with the production and dissemination of explanatory videos, to sensitize the health team to overcome this factor. A facilitator was the participation of some professionals who were part of the health team, who assumed the role of trusted collaborators and were in perfect communication with the research team, especially with regard to collecting fecal samples and ensuring their preservation until transport by those responsible for the research.

A protocol with a detailed description of the steps, rules of conduct, and recommendations regarding the collection, packaging, transport, and storage of fecal samples, with control of external factors that could eventually contaminate the sample, guaranteed the preservation and integrity of the DNA of the intestinal microbiota of preterm infants. Furthermore, the construction of an algorithm contributed to and facilitated the stages of the work process, and will certainly allow the study to be replicated in other health services, especially those that care for preterm infants.

## Authors’ contributions

Jessica Santos Passos Costa: Conception and design of the study, acquisition of data, analysis and interpretation of data and writing of the article.

Heli Vieira Brandão: Writing the article and final review of the manuscript.

Mara Viana Cardoso Amaral: Data acquisition and article writing.

Gabriela Cintra dos Santos: Data acquisition and article writing.

Camilla da Cruz Martins: Conception and design of the study, analysis, interpretation of data and writing of the article.

Michelle de Santana Xavier Ramos: Article writing and final review.

Tatiana de Oliveira Vieira: Conception and design of the study and writing of the article.

Raquel Guimarães Benevides: Conception and design of the study and writing of the article.

Graciete Oliveira Vieira: Conception and design of the study, analysis and interpretation of data and writing of the article.

## Funding

Conselho Nacional de Desenvolvimento Científico e Tecnológico. Edital Universal CNPq 28/2018.

## Declaration of competing interest

The authors declare no conflicts of interest.
